# Stable overexpression of miR-17 enhances recombinant protein production of CHO cells^☆^

**DOI:** 10.1016/j.jbiotec.2014.01.032

**Published:** 2014-04-10

**Authors:** Vaibhav Jadhav, Matthias Hackl, Gerald Klanert, Juan A. Hernandez Bort, Renate Kunert, Johannes Grillari, Nicole Borth

**Affiliations:** a Department of Biotechnology, University of Natural Resources and Life Sciences, Vienna, Austria; b Austrian Centre of Industrial Biotechnology GmbH, Graz, Austria

**Keywords:** Chinese hamster ovary (CHO) cells, microRNA (miRNA), miR-17, miR-92a;, miR17-92a cluster, Productivity

## Abstract

miRNAs negatively regulate gene expression at post-transcriptional level, and consequently play an important role in the control of many cellular pathways. The use of miRNAs to engineer Chinese hamster ovary (CHO) cells is an emerging strategy to improve recombinant protein production. Here, we describe the effect of transient and stable miRNA overexpression on CHO cell phenotype. Using an established transient miRNA screening protocol, the effects of miR-17, miR-92a and cluster miR17-92a on CHO growth and protein productivity were studied and followed by analysis of cell pools with stable overexpression of these miRNAs. CHO cells stably engineered with miR-17 exhibited both enhanced growth performance and a 2-fold increase in specific productivity, which resulted in a 3-fold overall increase in EpoFc titer. While further studies of miRNA–mRNA interactions will be necessary to understand the molecular basis of this effect, these data provide valuable evidence for miR-17 as a cell engineering target to enhance CHO cell productivity.

## Highlights

• Transient overexpression of miR-17 and miR-17–92 cluster enhanced growth rate.
• Biological effects of long term and stable overexpression of miRNAs in batch cultures were studied.
• Stable miR-17 engineered CHO cells had both improved growth rate and productivity.

## 1 Introduction

Recombinant production of therapeutic proteins has surpassed the 100 billion $ per year market volume and plays an important role in the global economy as well as in advanced medical care (Aggarwal, 2012; Brower, 2005). Today, a significant fraction of therapeutic proteins is produced in Chinese hamster ovary (CHO) cells due to their regulatory approval, biosafety compliance and ability to produce proteins with human-like glycosylation patterns. CHO cells inherently have high somatic genetic instability, which generates large clonal variation, a property that is commonly harnessed for screening, selection and development of production clones for expression of recombinant proteins (Hacker et al., 2009; Jayapal et al., 2007).

So far, several successful genetic engineering approaches have been used to enhance CHO cell performance in relation to apoptosis or autophagy, cell cycle (growth and proliferation), protein secretion and production, unfolded protein response (UPR) and metabolic engineering (Arden and Betenbaugh, 2006; Baker et al., 2001; Lee et al., 2013; Peng and Fussenegger, 2009; Wang et al., 2012; Wlaschin and Hu, 2007). In addition to engineering the expression of protein-coding genes, miRNAs have recently emerged as a tool to modify the phenotype of CHO cells (Barron et al., 2011b). RNAs are small non-coding RNA molecules, which negatively regulate gene expression at the post-transcriptional level. Similar to protein coding genes, transcription of most microRNAs is controlled by RNA polymerase II promoters. The longer primary transcripts undergo cleavage in the nucleus to form a hairpin like structure termed precursor microRNA (pre-miRNA) of 50–70 nucleotides length. Further, the pre-miRNA is exported from the nucleus to the cytoplasm where it is incorporated into a multimeric protein complex consisting of Dicer, Ago1–4 and others, and cleaved again to give rise to the miRNA induced silencing complex (miRISC) encompassing a 17–22 nt long mature miRNA. Subsequent sequence specific targeting of the miRISC toward the 3′-UTR of a target mRNA results either in the inhibition of mRNA translation or mRNA cleavage (Graves and Zeng, 2012; Havens et al., 2012). In recent years miRNA research has enhanced our understanding of gene expression control and lead to a better view of cellular physiology in both the normal as well as in a pathological state of a cell (Cui et al., 2006; Grillari et al., 2010; Ha, 2011). Evidence is accumulating for the great importance of small non-coding RNAs like piRNAs (Gerstl et al., 2013) and miRNAs in CHO cells for many fundamental biological processes including cell division and metabolism (Hatziapostolou et al., 2013), thus drawing the interest of cell engineers to this class of RNA molecules (Barron et al., 2011b; Hackl et al., 2012a; Jadhav et al., 2013; Muller et al., 2008).

To date, around 350 CHO miRNAs were sequenced (Hackl et al., 2011) and annotated (Hackl et al., 2012b). In view of the biotechnological use of miRNAs, the correlation of miRNA transcription to distinctive cellular phenotypes such as fast proliferation (Bort et al., 2012; Clarke et al., 2012) or stress response (Druz et al., 2012) was shown. In a recent study, the general importance of miRNA expression and the miRNA processing machinery in CHO cells was described (Hackl et al., 2014). These studies provide the basis for the development of CHO cell lines with improved phenotypes through engineered miRNA expression.

We have previously established a method for screening the effect of miRNA overexpression on growth and productivity of a recombinant EpoFc producing (EpoFc-CHO) model cell line (Jadhav et al., 2012) and observed a positive effect of transient miR-17 overexpression on CHO cell growth without negatively affecting cell specific productivity. A question remaining open with transient overexpression was whether such short term effects of increased miRNA expression remain valid in a cell line that continuously and stably overexpresses a miRNA. Here we therefore tested how the transient effects of the entire miR-17–92a cluster, miR-17, or miR-92a alone are consistent in stable overexpressing cell lines and how these growth enhancing miRNAs differ in their effects when overexpressed in recombinant EpoFc-CHO cell pools. Stable miR-17 overexpression confirmed a minor increase in growth rate, while at the same time resulted in 3-fold increase in EpoFc titers compared to controls. Overexpression of the entire miR-17–92a cluster resulted in no change in growth rate, but a reduced productivity, while miR-92a overexpression reduced growth and increased productivity. These results confirm that while transient over-expression is well able to identify engimiRs for stable miRNA engineering of CHO cells, it is still necessary to generate stable overexpressing cell lines for a detailed analysis of their effects. Our results also show that miR-17 is one of the so far few miRNAs demonstrated to boost productivity.

## 2 Materials and methods

### 2.1 Cell line and media

The recombinant CHO-EpoFc cell line was established as previously described (Lattenmayer et al., 2007) and was later adapted to growth in serum-free and l-glutamine free CD CHO medium (Gibco, Invitrogen, Carlsbad, CA, USA) supplemented with 0.096 μM MTX and 1 ml anti clumping agent (Gibco, Invitrogen, Carlsbad, CA, USA) per 500 ml medium (Taschwer et al., 2012). Cell cultures were cultivated in conical flasks with a working volume of 30 ml at 37 °C in a humidified atmosphere containing 7% carbon dioxide and constant shaking at 140 rpm.

### 2.2 Cloning miRNA expression plasmids

miRNA expression plasmids for miR-17 and miR-92a were developed as previously explained (Jadhav et al., 2012) by using sequences from miRbase: miR17 = MI0020419, miR-92a = MI0020560 (Kozomara and Griffiths-Jones, 2011). Briefly, chimeric CHO pre-miRNAs were cloned into pcDNA™6.2-GW/±EmGFP-miR vector (Invitrogen Inc., USA) in the 3′ untranslated (3′ UTR) region of the emerald green fluorescent protein (EmGFP) following the instruction manual. The miR17-92a cluster was cloned by PCR amplifying the Human genomic region (chr13: 92002682 + 92003780) containing the hsa-miR-17–92a cluster using primers FP: GGATCCCTAAATGGACCTCATATCTTTGAG and RP: GAATTCGAAAACAAGACAAGATGTATTTACAC and cloned into BamHI and EcoRI sites of pcDNA™6.2-GW/±EmGFP-miR vector. Sequences of all clones were confirmed using standard sequencing method.

### 2.3 Transient screening and generation of stable pools

The transient screening for candidate miRNA expression plasmids was carried out by standard protocol as previously described (Jadhav et al., 2012). Briefly, 1 × 10^7^ cells in exponential growth phase were transfected with 10 μg of pcDNA6.2-EmGFP-miR plasmid or pcDNA6.2-EmGFP-negative endo-free plasmid using the Nucleofector II (Lonza, Basel, Switzerland) and Nucleofector Kit V/program H-14. Post-transfection cells were seeded into 60 ml of pre-warmed culture medium in a conical flask. Cells were allowed to recover from electroporation in a static incubator at 37 °C for 2–3 h and then transferred to a humidified CO_2_ incubator with constant shaking at 140 rpm. Data for growth and productivity were recorded for the next four days.

For generation of pools with stable miRNA overexpression, cells were transferred 24 h post-transfection to 96-well culture plates containing 10,000 cells/well in selection media with 10 μg/ml Blasticidin-S (InvivoGen, California, USA). Cells were maintained by adding 50% fresh medium every 3–4 days for 4 weeks. Subsequently, surviving clones were expanded to 12-well plates for another 4 weeks with selection pressure and final pools were picked based on the GFP expression profiles. Most of the selected pools represented heterogeneous populations according to GFP expression. Thus, secondary selection was done without antibiotics selection pressure, but by sorting cells for homogenous GFP positive populations, followed by the generation of master cell banks that were used for further characterization and analysis.

### 2.4 Cell sorting and flow cytometry analysis

Cell sorting was done for viable and GFP positive cells in bulk using a FACS Vantage™ (Becton Dickinson, Franklin Lakes, NJ, USA), equipped with a Sort enhancement module. A combination of a SSC-H/FSC-H gates and GFP positive sorting gates was set using non-transfected cells. GFP positive cells were detected with a 488 nm argon ion laser and fluorescence was measured using a BP filter at 530/30 nm. Sorted cells (100,000) were collected in a 6-well plate with 3 ml pre-warmed culture medium supplemented with penicillin–streptomycin 1× concentration. After sorting, an aliquot of the sorted cells was run on the BD FACSCANTO to check the purity of the populations twice weekly for all pools for two weeks. Another round of sorting was done based on GFP expression once weekly for two weeks. Final pools deposited were homogenous and stable for GFP expression under normal culture conditions. All GFP measurements were performed using a BD FACS CANTO (Becton Dickinson, Franklin Lakes, NJ, USA) flow cytometer. A minimum of 10,000 events were analyzed at excitation wavelength of 488 nm using a 530/30 BP filter for collection of the emitted signal. Untransfected cells were used as negative control for gating of GFP-positive cells. Data were analyzed using the BD FACS DIVA software.

### 2.5 RNA isolation and quantitative real-time PCR

1–5 × 10^6^ cells were harvested and homogenized by vortexing in 1 ml Trizol reagent (Invitrogen, Carlsbad CA, USA) followed by 5-min incubation at room temperature and stored at −80 °C until used. Total RNA was isolated using the chloroform protocol provided by the manufacturer. In the final step RNA pellets were resuspended in 30 μl of nuclease free H_2_O. Absorbance at 230, 260 and 280 nm was measured using the ND-1000 spectrophotometer (NanoDrop Technologies, DE, USA) to calculate RNA quality and quantities. miRNA expression was measured using a TaqMan miRNA quantitative PCR assay from Applied Biosystems (Foster City, CA) that has been previously described (Cheng et al., 2005). Briefly, cDNA was made with 10 ng total RNA in 10 μl reactions using MultiScribe reverse transcriptase (Applied Biosystems, Foster City, CA, USA) and specific TaqMan^®^ primers for each miRNA (TaqMan^®^ miRNA Assay ID's, MIR92a: ID-00431, MIR17: ID-002308, MIR185: ID-002271). The cycle parameters for the RT reaction are 16 °C for 30 min, 42 °C for 30 min, 85 °C for 5 min, and hold at 4 °C. The PCR mix consists of the RT product, TaqMan^®^ 2× Universal PCR Master Mix and the appropriate 5× MicroRNA Assay Mix. The PCRs were run on the Corbett Rotorgene rotorcycler (Qiagen, Germany) including 4 technical replicates per sample. The expression of each miRNA relative to miR-185 (endogenous control) was determined using the ΔΔ*C*_t_ method. Average fold differences were calculated by normalizing the relative expression (Δ*C*_t_ values) to that in the negative control transfection. Average fold differences between batches are presented.

### 2.6 Analysis of growth and productivity

After selection of stable miRNA overexpressing pools their performance in batch culture was tested in small scale conical flasks. These batches were run in duplicate and repeated twice. Exponentially growing CHO-EpoFc cells were seeded in 60 ml of pre-warmed culture medium at a density of 2 × 10^5^ cells/ml. The daily measurements of cell viability and viable cell density were performed using a ViCell analyzer (Beckman Coulter, USA) based on the trypan-blue dye exclusion method. Total cell counts were analyzed using a Coulter Counter (Beckman Coulter, Fullerton, CA, USA) after isolation of nuclei in 0.1 M citric acid (2% Triton X-100) for at least 30 min. Growth data were further analyzed to calculate specific growth rates using the formula *μ* = (ln *X*_2_ − ln *X*_1_)/(*t*_2_ − *t*_1_) where *X*_1_ and *X*_2_ is viable cell number at the respective time points (*t*_1_ and *t*_2_). The EpoFc concentration was quantified by ELISA as described previously (Jadhav et al., 2012) using an ELISA reader (Sunrise, TECAN) at 492 nm and 620 nm as reference wavelength and the software Magellan according to the instruction manual. Specific productivity was calculated as *qP* = (*C*_*p*,*n*_ − *C*_*p*,1_)/CD where *C*_*p*_ is recombinant protein concentration at time *n* and CD is cumulative cell density, which is calculated as CD = ∑(*X*_*i*−1_ − *X*_*i*_)/*μ*, where *X*_*i*_ is integral viable cell density and *μ* is growth rate.

### 2.7 EpoFc gene copy number determination using real-time Q-PCR

Gene copy number determinations were made by quantitative PCR (Q-PCR). EpoFc PCR was performed with specific primers and as reference Bcl-2 was used, to develop a standard curve with data points ranging from 10^3^ to 10^8^ copies. Primers used for PCR were Bcl-2 FP: TTCAGCTCAAACTGGGCTTT, Bcl-2 RP: AACTTGAGCGGCTCCCTAAT, EpoFc FP: CATGGGGGTGCACGAATGTC, EpoFc RP: CAAGCTGCAGTGTTCAGCAC. The Q-PCR runs were done using the Corbett Rotorgene rotorcycler (Qiagen, Germany) system, amplification reactions (20 μl) were performed in 4 technical replicates per sample with 20 ng of input genomic DNA, 1 μl of each primer and 2 μl of 5× SensiMixPlus SYBR master mix. PCR parameters were as follows: an initial 10 min-denaturation step at 95 °C followed by 40 cycles of 15 s at 95 °C, 10 s at 65 °C and 15 s at 72 °C. Specificity of the primers was verified by a melting curve analysis of the PCR products with a temperature gradient of 0.2 °C/s from 68 °C to 98 °C. Copy number variation of EpoFc was determined as a ratio of the estimated copies of EpoFc gene to that of the reference gene, Bcl2.

## 3 Results

### 3.1 Effects of miRNA overexpression during transient transfection

**Fig. 1:**
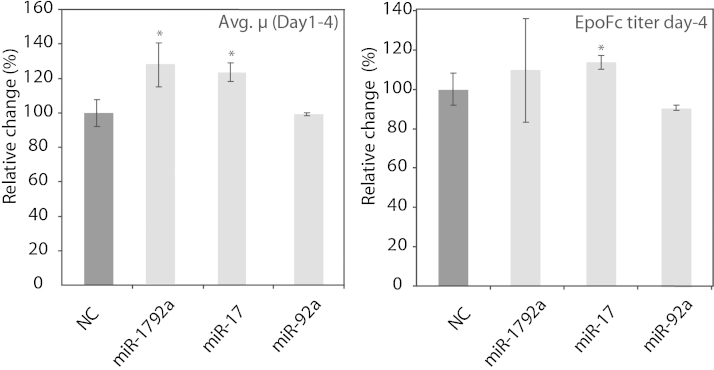
Effect of transient overexpression of miRNAs on growth rate and recombinant protein titers: (A) effect of miRNA overexpression on the growth rate is represented by the average growth rate (*μ*) calculated until day 4 of transient miRNA over-expression relative to the negative control transfection (NC). (B) Similarly, the effect on recombinant protein production is represented by the EpoFc titer on day 4 post-transfection. The data are presented as mean (±standard deviation; SD) of three independent experiments with two technical replicates each. *P < 0.05 to NC.

Previously, we proposed a screening platform for plasmid based, transient miRNA overexpression to test for biological effects that might change bio-industrially important cellular characteristics. Thereby we identified miR-17 as a promising engineering target as its transient overexpression increased cell proliferation (Jadhav et al., 2012). We extended our experiment by testing whether the whole miR-17–92 cluster, or the single miR miR-92 might have similar effects (Fig. 1). While miR-17 led to a 23 (±5)% and miR-17–92 cluster to a 27 (±12)% increase of mean growth rate (*μ*) over the period of 4 days, miR-92a did not show significant differences in *μ* when compared to the negative control (NC). Furthermore, miR-17 showed a moderate (13 ± 4.7%), but significant difference in the EpoFc titer. These results encouraged us to test the influence of these miRNAs in stable overexpression systems.

### 3.2 Generation and characterization of stable miRNA overexpressing pools

**Fig. 2:**
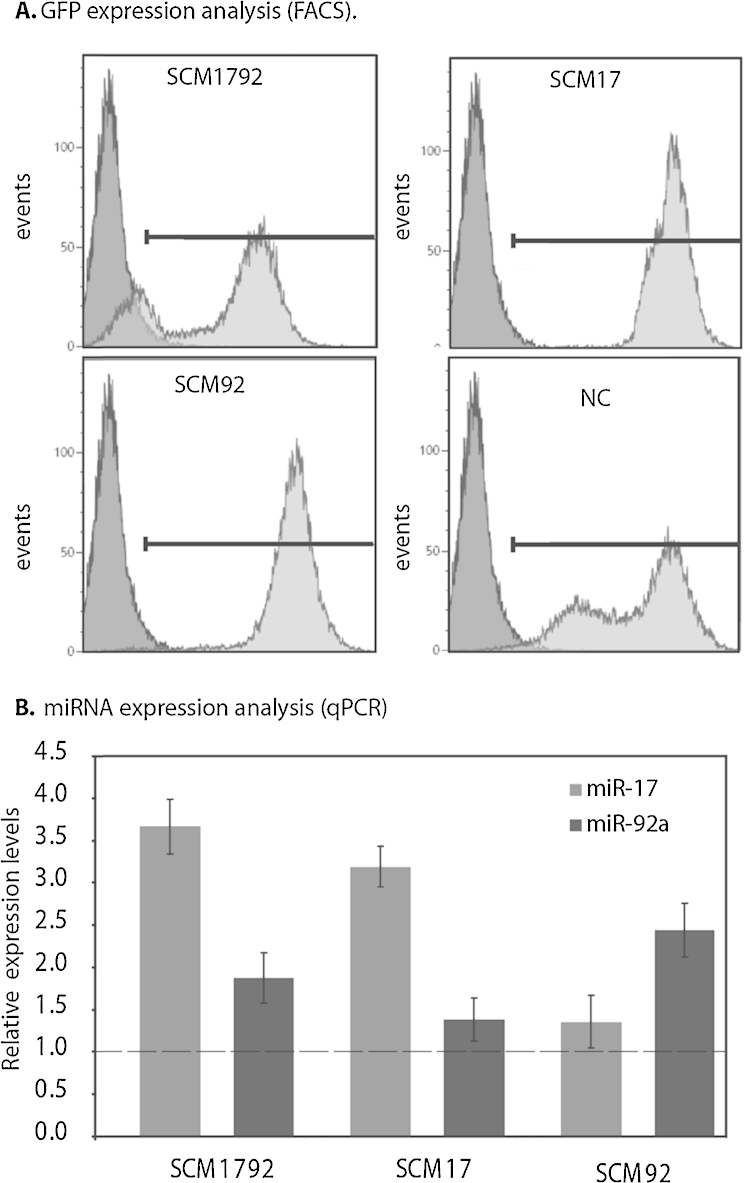
Selection and characterization of stable miRNA expression clones: miRNA overexpression was driven by a constitutive CMV promoter with co-expression of GFP. (A) The panel shows GFP-expression (Fl-1H) of 4 stable-pools overexpressing the miR-17–92 cluster (SCM-1792), miR-17 (SCM-17), miR-92a (SCM-92a) and the no-miR-vector control (NC) after the entire selection process. All graphs are overlaid with fluorescence of untransfected cells. (B) qPCR was performed to assess the miRNA overexpression. The expression of miR-17 and miR-92a relative to miR-185 expression (endogenous control) was determined using the ΔΔ*C*_t_ method. Average fold differences were calculated by normalizing the relative expression (Δ*C*_t_ values) to that of the negative control transfection.

The advantage of vector-based systems for functional miRNA screening is that the same vectors can immediately be used to generate stable miRNA overexpressing CHO cells. Using the mammalian selection marker gene present on the vector, stable miR-17, miR-92a and miR-17–92a cluster expressing pools were generated. Such pools were preferred to clonal populations in order to reduce the bias by the inherent clonal phenotypic variation observed in CHO cells (Pilbrough et al., 2009). Generation of stable pools was carried out in two-steps: first we performed antibiotic-selection with Blasticidin-S (10 μg/ml), followed by sorting for GFP positive cells using FACS to enrich the miRNA expressing population. GFP expressions of the final selected populations are shown in Fig. 2A. SCM-17 (SCM = stable cells expressing miRNA), SCM-92a and negative control cells (NC) exhibited >90% GFP positive cells, while for the miR-17–92a cluster (SCM-1792) we could only achieve 60% GFP enrichment even after two rounds of GFP cell sorting. Since GFP negative populations reappeared soon after each round of sorting, we speculate that GFP negative cells are a consequence of destabilized GFP translation due to the simultaneous cleavage of multiple miRNAs from the 3′ UTR of GFP. To assess the miRNA overexpression in these populations, total RNA was isolated from 10^6^ cells and miR-17, miR-92a and miR-185 (as reference gene) were analyzed using TaqMan real time qPCR assays. The fold changes relative to miR-185 were determined using the ΔΔ*C*_t_ method and compared to the NC population (Fig. 2B). For SCM-1792 cells the miR-17 and miR-92a levels were found to be 3.5- and 2.0-fold increase. In case of SCM-17 cells, miR-17 was 3.2-fold overexpressed, and as expected no significant overexpression was observed for miR-92a. Vice-versa, SCM-92a exhibited only miR-92a upregulation. Thus, the GFP and miRNA expression provided proof for successful stable overexpression of specific miRNAs in these populations.

### 3.3 The effects of stable miRNA overexpression on cell proliferation and recombinant protein production

**Fig. 3:**
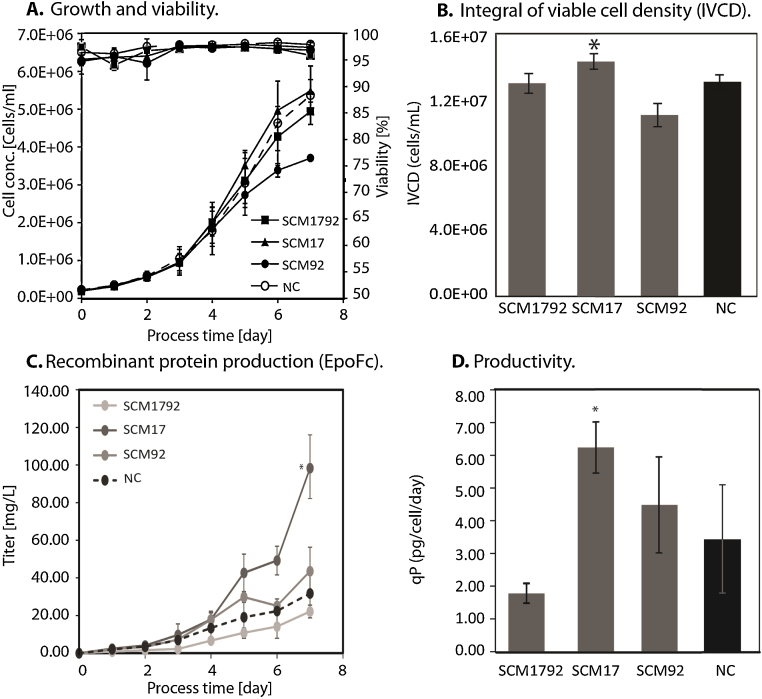
Analysis of culture performance of stable miRNA overexpressing EpoFc producers in shaken batch cultures: (A) mean viable cell density (primary *y*-axis) and viability (secondary *y*-axis) of three independent batches of SCM-1792(■), SCM-17(▴), SCM-92 (●), and NC (○). (B) Integral of viable cell density (IVCD) reached until day 7. (C) EpoFc (mg/L) concentrations during the batch, quantified by ELISA. (D) Average specific productivity (*qP*) calculated from day 1 to day 7 of the batch. The data are represented as mean (±standard deviation; SD) of three independent experiments with two technical replicates each. *P < 0.05 to NC.

Stably engineered cells were cultivated in batch cultures in shaker flasks to evaluate growth, viability and recombinant EpoFc production (Fig. 3). We observed that both SCM-1792 and SCM-17 showed a minor increase in growth rate during early exponential phase when compared to the NC population as indicated by specific growth rate analysis (data not shown). While SCM-17 cells sustained this faster growth until the end of the batch and therefore showed a significant increase in cumulative cell density of 15% (Fig. 3B), SCM-1792 cells entered stationary phase earlier than the control and therefore only reached a comparable cumulative cell density relative to NC. SCM-92a cells, on the other hand, had both a reduced growth and cumulative cell density.

The EpoFc protein titers in mg/L were observed to be significantly higher for SCM-17 cells (98.3 ± 25.3), compared to SCM-1792 (22.2 ± 3.4), SCM-92 (43.5 ± 12.8) and NC (31.8 ± 11.2) (Fig. 3C). While SCM-1792 cells exhibited even weaker titers and specific productivity compared to the negative control, SCM-92a cells produced equal amounts of EpoFc. Surprisingly, miR-17 overexpression in SCM-17 cells resulted in a 3-fold increase in titer compared to NC cells and a 2-fold increase in specific productivity (*qP*, Fig. 3D). Therefore, it seems that miR-17 overexpression not only increases cell proliferation but also modulates recombinant protein production in these cells.

**Fig. 4:**
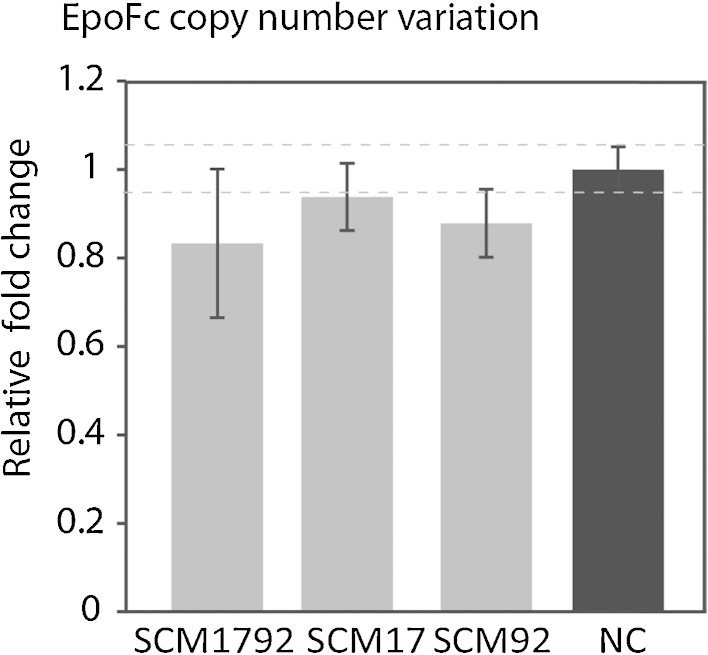
EpoFc gene copy number analysis: EpoFc gene copy numbers in the miRNA overexpressing pools were analyzed using real-time Q-PCR. Shown is the relative copy number of EpoFc relative to the reference gene, normalized to the negative control. The data are represented as mean (±standard deviation; SD) of four replicates.

To confirm that this increase in protein production is due to miR-17 overexpression and not due to other confounding factors such as selection of a variant population, we checked EpoFc gene copy numbers by real-time qPCR (Fig. 4). The results show no significant changes in EpoFc gene copy numbers across all four pools, thus confirming the positive effect of miR-17 overexpression on growth and especially recombinant protein production in CHO cells.

## 4 Discussion

In the past decade miRNA research has gained much attention in biology and found broad applications including diagnostics, therapeutics and cell engineering (Barron et al., 2011b; Bratkovic et al., 2012; Hackl et al., 2012a; Jadhav et al., 2013; Osman, 2012). Here we present results from both transient and stable overexpression of mi-17, miR-92a and the entire miR-17–92 cluster on cell proliferation and recombinant protein production in CHO cells. While transient screening is a fast approach to screen for promising candidates, one could argue that the effect of miRNA overexpression for a period of 3–5 days may be different from the effect generated by a continuous, long term increase in their transcription. Thus, the transient screening approach requires stable engineering of cells for validation of results and to ensure beneficial effects for industrial production of recombinant proteins. Our results show that the transient positive effects on growth could also be observed for the stably engineered CHO cell pools during early exponential culture, which is indeed the state that is best reflected by the transient protocol. During later culture phases, however, the effects became more divergent: while both miR92a and the cluster significantly reduced growth, resulting in a decreased (miR-92a) or comparable (miR-17–92a) IVCD relative to the control, miR-17 overexpressing cells continued to grow, resulting in an increased overall IVCD. A possible explanation for these diverging results is the fact that mRNA levels in cells during a batch culture are subject to continuous changes, as is the culture environment (Bort et al., 2012). Thus, constitutive miRNA overexpression could result in the repression of new targets as cell behavior changes during batch-cultivation and resulting in a “new” effect different to that observed during the short transient testing phase. Nevertheless, initial transient testing of miRNA effects enables time-efficient pre-selection of promising candidates, specifically if growth rate is a major target for engineering as the set-up of the transient screening best mirrors early exponential growth phase. In case other culture phases are of interest (for instance increasing productivity during stationary phase) the protocol for transient screening would have to be adapted accordingly.

Unexpectedly, stable overexpression of miR-17 not only enhanced growth, but also specific protein productivity (2-fold), resulting in a 3-fold increase in titer. This is quite remarkable in the context of cell engineering, since commonly growth and specific productivity are inversely correlated to a certain extent. This has been shown by both physical (temperature) and genetic (coding and non-coding) manipulations of CHO cells, which either reduce or arrest growth, facilitating enhanced specific productivity (Barron et al., 2011a; Fogolin et al., 2004; Kaufmann et al., 2001; Sunley and Butler, 2010; Yoon et al., 2003). Stable overexpression of miR-17, however, slightly increases growth but at the same time significantly enhances productivity. This parallel induction of both cell-specific growth rate and productivity is unique and requires a more detailed investigation of the precise interactions and effects caused by miR-17. So far, literature indicates that the six mature miRNAs derived from the cluster play a role in several hematopoietic malignancies, solid tumors and lung carcinoma (Olive et al., 2010) and in B lymphomagenesis (He et al., 2005) and that these miRNAs act as important components of the pathways that regulate many genes involved in G1/S-phase cell cycle. This would explain their significant roles during tumor development and tumor maintenance (Yang et al., 2013). It has been shown that miR-17–92 can target several other cellular pathways (e.g., Wnt, Jak/Stat signaling and TGF-β pathway) apart from cell cycle and apoptosis (Doebele et al., 2010; Mestdagh et al., 2010; Uziel et al., 2009). Further, transcriptomic analysis of CHO clones exhibiting different growth rates (Clarke et al., 2012) indicates a potential role of this cluster in growth and recombinant protein production. Importantly, miR-17 was shown to target TBC1D2/Armus, which plays an important role in membrane trafficking (Serva et al., 2012) and could therefore enhance protein secretion.

With respect to investigating miRNA function in production relevant CHO cell lines, one needs to be aware that it is highly dependent on cell type and the cell-specific transcriptome, respectively. It is therefore not clear to what extent results obtained with human or mouse (tumor) cell lines may be relevant for CHO. For a more reliable prediction of miRNA function in CHO cells, miRNA:mRNA target databases specifically designed for CHO are urgently required. This includes thorough annotation of mRNA untranslated regions (UTR) in the CHO genome database (Hammond et al., 2012). For specific cases like the presented one, the analysis of the transcriptome as well as proteome of miRNA-engineered cell lines is required to analyze direct effects of miRs on protein expression during each stage of the batch culture, while methods like TAP-tar (Nonne et al., 2010) will identify direct miRNA:mRNA interactions. Both of these approaches are currently underway.

In conclusion, this is one of the first reports describing stable overexpression of miRNAs in CHO cells and analyzing its effects on growth and recombinant protein production. Further analysis of these stable miRNA-expressing CHO cells will provide molecular insights eventually leading to refined miRNA engineering strategies for CHO cells.

## Conflict of interest

JG is co-founder of Evercyte.

## References

[bib0005] Aggarwal S.R. (2012). What's fueling the biotech engine – 2011 to 2012. Nature Biotechnology.

[bib0010] Arden N., Betenbaugh M.J. (2006). Regulating apoptosis in mammalian cell cultures. Cytotechnology.

[bib0015] Baker K.N., Rendall M.H., Hills A.E., Hoare M., Freedman R.B., James D.C. (2001). Metabolic control of recombinant protein N-glycan processing in NS0 and CHO cells. Biotechnology and Bioengineering.

[bib0020] Barron N., Kumar N., Sanchez N., Doolan P., Clarke C., Meleady P., O'Sullivan F., Clynes M. (2011). Engineering CHO cell growth and recombinant protein productivity by overexpression of miR-7. Journal of Biotechnology.

[bib0025] Barron N., Sanchez N., Kelly P., Clynes M. (2011). MicroRNAs: tiny targets for engineering CHO cell phenotypes?. Biotechnology Letters.

[bib0030] Bort J.A.H., Hackl M., Hoflmayer H., Jadhav V., Harreither E., Kumar N., Ernst W., Grillari J., Borth N. (2012). Dynamic mRNA and miRNA profiling of CHO-K1 suspension cell cultures. Biotechnology Journal.

[bib0035] Bratkovic T., Glavan G., Strukelj B., Zivin M., Rogelj B. (2012). Exploiting microRNAs for cell engineering and therapy. Biotechnology Advances.

[bib0040] Brower A. (2005). Number of monoclonal antibodies on market nearly doubles by 2008. Biotechnology Healthcare.

[bib0045] Cheng A.M., Byrom M.W., Shelton J., Ford L.P. (2005). Antisense inhibition of human miRNAs and indications for an involvement of miRNA in cell growth and apoptosis. Nucleic Acids Research.

[bib0050] Clarke C., Henry M., Doolan P., Kelly S., Aherne S., Sanchez N., Kelly P., Kinsella P., Breen L., Madden S.F., Zhang L., Leonard M., Clynes M., Meleady P., Barron N. (2012). Integrated miRNA, mRNA and protein expression analysis reveals the role of post-transcriptional regulation in controlling CHO cell growth rate. BMC Genomics.

[bib0055] Cui Q., Yu Z., Purisima E.O., Wang E. (2006). Principles of microRNA regulation of a human cellular signaling network. Molecular Systems Biology.

[bib0060] Doebele C., Bonauer A., Fischer A., Scholz A., Reiss Y., Urbich C., Hofmann W.K., Zeiher A.M., Dimmeler S. (2010). Members of the microRNA-17-92 cluster exhibit a cell-intrinsic antiangiogenic function in endothelial cells. Blood.

[bib0065] Druz A., Betenbaugh M., Shiloach J. (2012). Glucose depletion activates mmu-miR-466h-5p expression through oxidative stress and inhibition of histone deacetylation. Nucleic Acids Research.

[bib0070] Fogolin M.B., Wagner R., Etcheverrigaray M., Kratje R. (2004). Impact of temperature reduction and expression of yeast pyruvate carboxylase on hGM-CSF-producing CHO cells. Journal of Biotechnology.

[bib0075] Gerstl M.P., Hackl M., Graf A.B., Borth N., Grillari J. (2013). Prediction of transcribed PIWI-interacting RNAs from CHO RNAseq data. Journal of Biotechnology.

[bib0080] Graves P., Zeng Y. (2012). Biogenesis of mammalian microRNAs: a global view. Genomics, Proteomics & Bioinformatics.

[bib0085] Grillari J., Hackl M., Grillari-Voglauer R. (2010). miR-17–92 cluster: ups and downs in cancer and aging. Biogerontology.

[bib0090] Ha T.Y. (2011). MicroRNAs in human diseases: from cancer to cardiovascular disease. Immune Network.

[bib0095] Hacker D.L., De Jesus M., Wurm F.M. (2009). 25 years of recombinant proteins from reactor-grown cells – where do we go from here?. Biotechnology Advances.

[bib0100] Hackl M., Borth N., Grillari J. (2012). miRNAs – pathway engineering of CHO cell factories that avoids translational burdening. Trends in Biotechnology.

[bib0105] Hackl M., Jadhav V., Jakobi T., Rupp O., Brinkrolf K., Goesmann A., Puhler A., Noll T., Borth N., Grillari J. (2012). Computational identification of microRNA gene loci and precursor microRNA sequences in CHO cell lines. Journal of Biotechnology.

[bib3160] Hackl M., Jadhav V., Klanert G., Karbiener M., Scheideler M., Grillari J., Borth N. (2014). Analysis of microRNA transcription and post-transcriptional processing by Dicer in the context of CHO cell proliferation. Journal of Biotechnology.

[bib0110] Hackl M., Jakobi T., Blom J., Doppmeier D., Brinkrolf K., Szczepanowski R., Bernhart S.H., Siederdissen C.H., Bort J.A., Wieser M., Kunert R., Jeffs S., Hofacker I.L., Goesmann A., Puhler A., Borth N., Grillari J. (2011). Next-generation sequencing of the Chinese hamster ovary microRNA transcriptome: Identification, annotation and profiling of microRNAs as targets for cellular engineering. Journal of Biotechnology.

[bib0115] Hammond S., Kaplarevic M., Borth N., Betenbaugh M.J., Lee K.H. (2012). Chinese hamster genome database: an online resource for the CHO community at www.CHOgenome.org. Biotechnology and Bioengineering.

[bib0120] Hatziapostolou M., Polytarchou C., Iliopoulos D. (2013). miRNAs link metabolic reprogramming to oncogenesis. Trends in Endocrinology and Metabolism.

[bib0125] Havens M.A., Reich A.A., Duelli D.M., Hastings M.L. (2012). Biogenesis of mammalian microRNAs by a non-canonical processing pathway. Nucleic Acids Research.

[bib0130] He L., Thomson J.M., Hemann M.T., Hernando-Monge E., Mu D., Goodson S., Powers S., Cordon-Cardo C., Lowe S.W., Hannon G.J., Hammond S.M. (2005). A microRNA polycistron as a potential human oncogene. Nature.

[bib0135] Jadhav V., Hackl M., Bort J.A., Wieser M., Harreither E., Kunert R., Borth N., Grillari J. (2012). A screening method to assess biological effects of microRNA overexpression in Chinese hamster ovary cells. Biotechnology and Bioengineering.

[bib0140] Jadhav V., Hackl M., Druz A., Shridhar S., Chung C.Y., Heffner K.M., Kreil D.P., Betenbaugh M., Shiloach J., Barron N., Grillari J., Borth N. (2013). CHO microRNA engineering is growing up: recent successes and future challenges. Biotechnology Advances.

[bib0145] Jayapal K.R., Wlaschin K.F., Hu W.S., Yap M.G.S. (2007). Recombinant protein therapeutics from CHO cells – 20 years and counting. Chemical Engineering Progress.

[bib0150] Kaufmann H., Mazur X., Marone R., Bailey J.E., Fussenegger M. (2001). Comparative analysis of two controlled proliferation strategies regarding product quality, influence on tetracycline-regulated gene expression, and productivity. Biotechnology and Bioengineering.

[bib0155] Kozomara A., Griffiths-Jones S. (2011). miRBase: integrating microRNA annotation and deep-sequencing data. Nucleic Acids Research.

[bib2160] Lattenmayer C., Loeschel M., Schriebl K., Steinfellner W., Sterovsky T., Trummer E., Vorauer-Uhl K., Müller D., Katinger H., Kunert R. (2007). Protein-free transfection of CHO host cells with an IgG-fusion protein: selection and characterization of stable high producers and comparison to conventionally transfected clones. Biotechnology and Bioengineering.

[bib0160] Lee K.H., Tsutsui T., Honda K., Asano R., Kumagai I., Ohtake H., Omasa T. (2013). Generation of high-producing cell lines by overexpression of cell division cycle 25 homolog A in Chinese hamster ovary cells. Journal of Bioscience and Bioengineering.

[bib0165] Mestdagh P., Bostrom A.K., Impens F., Fredlund E., Van Peer G., De Antonellis P., von Stedingk K., Ghesquiere B., Schulte S., Dews M., Thomas-Tikhonenko A., Schulte J.H., Zollo M., Schramm A., Gevaert K., Axelson H., Speleman F., Vandesompele J. (2010). The miR-17–92 microRNA cluster regulates multiple components of the TGF-beta pathway in neuroblastoma. Molecular Cell.

[bib0170] Muller D., Katinger H., Grillari J. (2008). MicroRNAs as targets for engineering of CHO cell factories. Trends in Biotechnology.

[bib0175] Nonne N., Ameyar-Zazoua M., Souidi M., Harel-Bellan A. (2010). Tandem affinity purification of miRNA target mRNAs (TAP-Tar). Nucleic Acids Research.

[bib0180] Olive V., Jiang I., He L. (2010). miR-17–92, a cluster of miRNAs in the midst of the cancer network. The International Journal of Biochemistry & Cell Biology.

[bib0185] Osman A. (2012). MicroRNAs in health and disease – basic science and clinical applications. Clinical Laboratory.

[bib0190] Peng R.W., Fussenegger M. (2009). Molecular engineering of exocytic vesicle traffic enhances the productivity of Chinese hamster ovary cells. Biotechnology and Bioengineering.

[bib0195] Pilbrough W., Munro T.P., Gray P. (2009). Intraclonal protein expression heterogeneity in recombinant CHO cells. PLoS ONE.

[bib0205] Serva A., Knapp B., Tsai Y.T., Claas C., Lisauskas T., Matula P., Harder N., Kaderali L., Rohr K., Erfle H., Eils R., Braga V., Starkuviene V. (2012). miR-17-5p regulates endocytic trafficking through targeting TBC1D2/Armus. PLOS ONE.

[bib0210] Sunley K., Butler M. (2010). Strategies for the enhancement of recombinant protein production from mammalian cells by growth arrest. Biotechnology Advances.

[bib0215] Taschwer M., Hackl M., Hernandez Bort J.A., Leitner C., Kumar N., Puc U., Grass J., Papst M., Kunert R., Altmann F., Borth N. (2012). Growth, productivity and protein glycosylation in a CHO EpoFc producer cell line adapted to glutamine-free growth. Journal of Biotechnology.

[bib0220] Uziel T., Karginov F.V., Xie S., Parker J.S., Wang Y.D., Gajjar A., He L., Ellison D., Gilbertson R.J., Hannon G., Roussel M.F. (2009). The miR-17–92 cluster collaborates with the Sonic Hedgehog pathway in medulloblastoma. Proceedings of the National Academy of Sciences of the United States of America.

[bib0225] Wang Z., Ma X., Fan L., Rhee W.J., Park T.H., Zhao L., Tan W.S. (2012). Understanding the mechanistic roles of 30Kc6 gene in apoptosis and specific productivity in antibody-producing Chinese hamster ovary cells. Applied Microbiology and Biotechnology.

[bib0230] Wlaschin K.F., Hu W.S. (2007). Engineering cell metabolism for high-density cell culture via manipulation of sugar transport. Journal of Biotechnology.

[bib0235] Yang L., Meng Y., Bao C., Liu W., Ma C., Li A., Xuan Z., Shan G., Jia Y. (2013). Robustness and backbone motif of a cancer network regulated by miR-17–92 cluster during the G1/S transition. PLOS ONE.

[bib0240] Yoon S.K., Song J.Y., Lee G.M. (2003). Effect of low culture temperature on specific productivity, transcription level, and heterogeneity of erythropoietin in Chinese hamster ovary cells. Biotechnology and Bioengineering.

